# Optical Bionanosensors for Sepsis Diagnostics

**DOI:** 10.1002/smll.202409042

**Published:** 2025-01-02

**Authors:** Christina Derichsweiler, Svenja Herbertz, Sebastian Kruss

**Affiliations:** ^1^ Biomedical Nanosensors Fraunhofer Institute for Microelectronic Circuits and Systems Finkenstrasse 61 47057 Duisburg Germany; ^2^ Physical Chemistry Ruhr‐University Bochum Universitätsstrasse 150 44801 Bochum Germany

**Keywords:** biosensors, diagnostics, nanomaterials, optical, sepsis

## Abstract

Sepsis is a global health challenge, characterized by a dysregulated immune response, leading to organ dysfunction and death. Despite advances in medical care, sepsis continues to claim a significant toll on human lives, with mortality rates from 10–25% for sepsis and 30–50% for septic shock, making it a leading cause of death worldwide. Current diagnostic methods rely on clinical signs, laboratory parameters, or microbial cultures and suffer from delays and inaccuracies. Therefore, there is a pressing need for novel diagnostic tools that can rapidly and accurately identify sepsis. This review highlights advances in biosensor development that could ultimately lead to faster and more accurate sepsis diagnostics. The focus is on nanomaterial‐based optical approaches that promise rapid diagnostics without the need for large equipment or trained personnel. An overview of sepsis is provided, highlighting potential molecular targets and the challenges they present for assay development. The requirements for an ideal point‐of‐care test (POC) are discussed, including speed, simplicity, and cost‐effectiveness. Different nanomaterials suitable for various optical detection methods are reviewed and innovative nanosensors are discussed for sepsis diagnostics, focusing on chemical design and approaches to increase selectivity by multiplexing.

## Graphical abstract

Sepsis is a global health issue triggered by an uncontrolled immune response to infections, causing organ failure and high mortality. Despite advances in medical care, traditional diagnostic methods are still not sufficient. This review highlights recent advancements in nanomaterial‐based optical biosensors for rapid sepsis diagnostics, discussing molecular targets, challenges in chemical nanosensor design, and essential requirements for effective point‐of‐care tests.

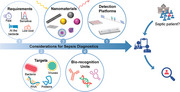

## 1 Introduction

### 1.1 Clinical Relevance of Sepsis

Although sepsis has been known for more than 100 years and was even classified as a global threat by the WHO in 2017, it is still one of the leading causes of death.^[^ ^1^, ^2^ ^]^ Worldwide, one in five deaths is related to sepsis (11 million people per year), and around 50 million people suffer from sepsis every year (status 1990–2017).^[^ ^3^, ^4^ ^]^ Sepsis, also known as blood poisoning, is defined as “life‐threatening organ dysfunction caused by a dysregulated host response to infection.”^[^ ^5^ ^]^ If sepsis is accompanied by circulatory and metabolic disorders and the patient has persistent hypotension, this condition is referred to as septic shock.^[^ ^5^ ^]^ In this state, the mortality of the patient rapidly increases.

Assessing mortality in sepsis is difficult. For example, data collection in low‐ and middle‐income countries might not be as detailed as in high‐income countries.^[^ ^6^, ^7^ ^]^ Also, in the case of people with chronic diseases such as cancer, the underlying disease is sometimes given as the cause of death rather than sepsis, which also distorts the results.^[^ ^8^ ^]^ Studies revealed that mortality in general also depends on whether the country has a low, medium, or high income.^[^ ^9^ ^]^ Mortality is therefore linked to various aspects and the range of 10–25% for sepsis and 30–50% for septic shock, shows that an exact determination is not possible.^[^ ^8^ ^]^

The costs for sepsis patients vary based on severity and country.^[^ ^10^, ^11^ ^]^ Delayed diagnosis results in higher treatment expenses. In 2013, the Federal Insurance Office estimated the average cost of a sepsis patient in Germany at 27,468 €, which means a total of 7.7 billion € for all patients.^[^ ^12^ ^]^ In the United States, total hospital costs for sepsis were estimated at $23.7 billion in 2013 and $52.1 billion in 2021.^[^ ^13^, ^14^ ^]^ Therefore, sepsis is a huge burden for global healthcare systems.

Anyone can be affected by sepsis, but people aged 65 or older, as well as children younger than one year, are at higher risk.^[^ ^15^ ^]^ Age and overall health status are critical factors. People with chronic diseases such as diabetes, HIV/AIDS, hepatitis, and cancer, or those who have already survived sepsis, are more susceptible.^[^ ^16^ ^]^ A weakened immune system further increases this risk.^[^ ^17^ ^]^ Cancer patients, for example, are at higher risk of being affected by sepsis due to cancer treatments like chemotherapy. Furthermore, long‐term catheterization, venous access, or drainage also make them more susceptible to sepsis, as they increase the risk of infection.^[^ ^18^ ^]^

The most common causes of sepsis are infections from bacteria, which can be Gram‐positive or Gram‐negative. Infections caused by viruses and fungi can also cause sepsis, but less frequently.^[^ ^19^ ^]^ Both the different pathogens and the fact that any infection can potentially develop into sepsis contribute to the complexity of this disease. However, some sites of infection are particularly susceptible, such as the respiratory tract, skin, abdomen, central nervous system, and urinary tract.^[^ ^20^, ^21^, ^22^, ^23^ ^]^

### 1.2 Challenges in Defining and Diagnosing Sepsis

**Figure 1:**
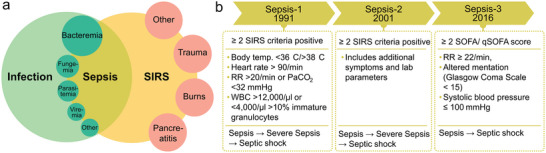
Sepsis as a disease. a) Relationship between Sepsis, Systemic Inflammatory Response Syndrome (SIRS) and infections. Adapted with permission.^[^ ^24^ ^]^ Copyright 1992, Elsevier. The size of the inner circles indicates the probability that a disease is caused by a specific pathogen. b) Summary of the different clinical definitions of sepsis (respiratory rate (RR), white blood cell count (WBC), sequential organ failure assessment score (SOFA), quickSOFA score (qSOFA)).^[^ ^5^, ^24^, ^25^ ^]^

In 1991, the first definition of sepsis was established, together with a more precise definition of Systemic Inflammatory Response Syndrome (SIRS) and the formulation of its diagnostic criteria. While SIRS characterize an inflammatory process of unknown origin, sepsis occurs when two or more SIRS criteria are met in conjunction with the presence of an infection. Within this context, sepsis was further categorized into three distinct classifications: sepsis, severe sepsis, and septic shock. The relation between these diagnoses is shown in **Figure** **1**a.^[^ ^24^ ^]^ Growing knowledge led to several changes in the definition to facilitate the identification. In 2001, the definition was revised, focusing on refining the list of signs and symptoms.^[^ ^25^ ^]^ The criticism of definitions based on SIRS is that while sensitivity is high, specificity is low.^[^ ^26^ ^]^ As a result, the definition was revised in 2016, leading to the Sepsis‐3 definition, which is based on the Sepsis‐related Organ Failure Assessment (SOFA) score instead of SIRS criteria.^[^ ^5^, ^27^, ^28^ ^]^ The SOFA score, introduced in 1994, serves as a guideline for assessing organ dysfunction in six organs, ranking functionality from 1 (normal) to 4 (most abnormal).^[^ ^29^ ^]^ According to Sepsis‐3, organ dysfunction is present if the SOFA score increases by more than two points. In the revised definition, severe sepsis was removed, and a distinction was made only between sepsis and septic shock. Since identifying sepsis based on the SOFA score requires laboratory tests, the Sepsis‐3 task force defined the quickSOFA (qSOFA) score, focusing on three parameters: systolic blood pressure (SBP≤100 mmHg), respiratory rate (≥22 breaths per min), and altered mentation (Glasgow coma scale <5).^[^ ^5^ ^]^ The qSOFA score is intended for rapid assessment in everyday clinical practice and enables a more precise evaluation in follow‐up examinations. Each new definition attempts to clarify and specify the definition of sepsis and to facilitate the identification of affected patients. In 2021, the Surviving Sepsis Campaign stated that neither the qSOFA score nor the SIRS criteria are sufficient as the sole tool for recognizing sepsis. The qSOFA score was found to have better specificity, but lower sensitivity.^[^ ^30^ ^]^ All these discussions show that considerable advances are still needed in the diagnosis of sepsis.

### 1.3 Sepsis Mortality: A Matter of Time

One of the main challenges of sepsis is that the success of treatment is time sensitive. Although various pathogens can cause sepsis, the typical first step is administration of broad‐spectrum antibiotics. The survival rate of patients correlates with the time until administration of effective antimicrobial therapy.^[^ ^31^ ^]^ Early administration significantly reduces the mortality, as shown by Ferrer et al.,^[^ ^32^ ^]^ who analyzed 28 150 patients from 165 intensive care units (ICU), who developed severe sepsis or septic shock. They analyzed the changes in mortality depending on the time of antibiotic administration and found that rapid and targeted treatment significantly increases the chances of recovery. As time is a critical factor, patients with suspected sepsis are given broad‐spectrum antibiotics before the test results determine the most effective therapy. This approach is intended to offer the best chances of recovery, but also harbors risks such as increased resistance and possible side effects of antibiotics.^[^ ^19^, ^33^ ^]^

## 2 Sepsis Biomarkers

Biomarkers provide diagnostic, prognostic, predictive, and pharmacodynamic/therapeutic information. Diagnostic biomarkers identify the presence and type of a disease, such as whether sepsis is viral or bacterial. Prognostic biomarkers predict the outcome and the course of a given disease. In comparison, predictive biomarkers determine which treatments are likely to be effective, while pharmacodynamic/therapeutic biomarkers predict drug responses and the efficacy of a therapy.^[^ ^34^, ^35^, ^36^ ^]^

In contrast to other diseases, sepsis does not have a single or standard biomarker, as is the case with heart attacks (troponin I) or diabetes (glucose).^[^ ^37^, ^38^ ^]^ The ideal biomarker should distinguish between an infected and a non‐infected person, identify the origin of the infection (viral or bacterial), and assess the severity and outcome. One reason why there is no ideal single biomarker for sepsis is that complex cellular interactions and a variety of different molecules are involved. However, these molecules are not only specific to sepsis but may also be present in other clinical conditions.^[^ ^39^, ^40^, ^41^ ^]^ More than 250 different biomarkers for sepsis have been identified so far.^[^ ^42^ ^]^ Several comprehensive review articles address this topic.^[^ ^39^, ^42^, ^43^, ^44^ ^]^ Here, we focus on a few that are of interest for nanomaterial‐based sensors (**Table** **1** **)**. Diagnostics based on biomarkers can be approached in two ways: one uses single biomarker detection while the other detects multiple parameters. Some studies conclude that combining different biomarkers can be beneficial, so biomarker‐based diagnostic tools with a multiplexing approach are expected to be the way forward.^[^ ^40^, ^45^, ^46^ ^]^

The most clinically relevant biomarkers are C‐reactive protein (CRP), interleukin‐6 (IL‐6), and procalcitonin (PCT), whose serum concentrations increase in sepsis.^[^ ^47^, ^48^ ^]^ CRP is known for its high sensitivity, but low specificity for sepsis identification, as its levels are also influenced by chronic conditions such as autoimmune diseases or by other triggers (e.g., trauma).^[^ ^49^, ^50^, ^51^, ^52^ ^]^ By measuring the kinetics of the CRP level, conclusions can be drawn about the course of the disease. In comparison, PCT levels rise following a bacterial infection. PCT has also been shown to indicate the severity of sepsis and has significant potential for antibiotic stewardship in respiratory tract infections, as its levels decrease during recovery.^[^ ^53^, ^54^ ^]^ IL‐6 is a pro‐inflammatory cytokine produced by leukocytes, making it a direct inflammatory marker. However, studies have shown that IL‐6 is only moderately effective in differentiating between sepsis and non‐sepsis. Nevertheless, IL‐6 is considered to have prognostic value.^[^ ^55^, ^56^, ^57^ ^]^ Lactate is another marker commonly evaluated in clinical practice. Like many other biomarkers, it cannot provide a definite statement about the presence of sepsis on its own, but in combination with other markers, it can make a helpful contribution.^[^ ^30^ ^]^ Studies revealed that lactate levels indicate patient mortality, thereby providing prognostic value.^[^ ^58^, ^59^ ^]^

**Table 1:** This table summarizes various studies that provide a general assessment of the properties of the highlighted biomarkers for different applications. However, the individual studies differ in terms of the type of patient samples used and the demographic characteristics of the patients. In addition, standardized cut‐off values are often not available. This table should be considered as an approximate reference for biomarkers and approximate quantities. It also shows the difficulty to identify quantitative biomarkers.

Biomarker	Half‐life	Type of biomarker	Cut‐off value	Parameters	Sensitivity Specificity	Refs.
C‐reactive protein (CRP)	Plasma: ≈9 h^[^ ^60^ ^]^	Diagnostic sepsis	12.00–90.00 mg L^−1^	Pooled study: Nine studies 1368 patients 495 septic 873 non‐septic	80.0% 61.0%	[61]
		Prognostic	> 62.8 mg L^−1^	One study: 813 patients 692 survivors 121 non‐survivors	52.1% 76.7%	[62]
Procalcitonin (PCT)	Serum: 25–30 h^[^ ^63^ ^]^	Diagnostic Sepsis	Not mentioned	Pooled study: 54 articles	82.0% 78.0%	[64]
		Diagnostic Sepsis	Between >0.28 and >0.5 ng mL^−1^	Pooled study: 19 studies 3012 patients	80.0% 75.0%	[65]
Interleukin‐6 (IL‐6)	Serum: ≈15 h^[^ ^66^ ^]^	Diagnostic Sepsis	Not mentioned	Pooled study: 54 articles	72.0% 70.0%	[64]
		Diagnostic Sepsis	52.60 pg mL^−1^	142 patients 51 with sepsis 46 with septic shock 45 as controls	80.4% 88.9%	[56]
		Diagnostic Septic shock	348.92 pg mL^−1^		76.1% 78.4%	
Lactate	Blood: ≈20 min^[^ ^67^ ^]^	Diagnostic Sepsis	1.6−2.5 mmol L^−1^	Surviving sepsis campaign 2021	66.0−83.0% 80.0−85.0%	[30]
		Prognostic	>2.5–≥4 mmol L^−1^	Summary of 3 studies 955 patients	65.6% 65.8%	[68, 69, 70]
Pentraxin 3 (PTX3)	Not reported^[^ ^71^ ^]^	Diagnostic Sepsis	5 ng mL^−1^	290 patients 213 ICU patients with sepsis and septic shock 77 healthy controls	98.0% 79.0%	[72]
		Diagnostic Septic shock	9 ng mL^−1^		93.0% 45.0%	[72]
		Prognostic	26.90 ng mL^−1^	160 patients 78 with sepsis 82 with septic shock	88.9% 49.5%	[73]
Heparin‐binding protein (HBP)	Plasma: 1–2 h^[^ ^74^ ^]^	Diagnostic Sepsis	≥28.1 ng mL^−1^	125 patients: 32 with sepsis 93 without sepsis	84.9% 78.3%	[75]
		Diagnostic Septic shock	≥103.5 ng mL^−1^		67.6% 82.1%	[75]
		Prognostic	161.415 ng mL^−1^	Pooled study: 28 studies 5508 patients	72.0% 72.0%	[76]
Neutrophil cluster of differentiation 64 (nCD64)	6 h^[^ ^77^ ^]^	Diagnostic Sepsis	Not mentioned	Pooled study: 54 articles	88.0% 88.0%	[64]
		Diagnostic Sepsis	Not mentioned	Pooled study: 14 studies 2471 patients: 1304 with sepsis 1167 controls	87.0% 89.0%	[78]
		Prognostic	Increase of 11% from day 0 to day 8	60 patients: 37 survivors 23 non‐survivors	87.5% 58.0%	[79]
Soluble triggering receptor expressed on myeloid cells‐1 (sTREM‐1)	Stable in blood with a short half‐life in vivo^[^ ^80^ ^]^	Diagnostic: Sepsis	30–60 000 pg mL^−1^	Pooled study: 17 studies 2263 patients: 1394 with sepsis 869 SIRS patients	85.0% 79.0%	[81]
		Diagnostic Sepsis	30–60 ng mL^−1^	Pooled study: 19 studies 2418 patients	82.0% 81.0%	[82]
		Prognostic	30–60 000 pg mL^−1^	Pooled study: 5 studies 423 patients: 291 survivors 132 non‐survivors	80.0% 75.0%	[81]
Circulating peptidase 3 (cDPP3)	20–70 min^[^ ^83^, ^84^ ^]^	Prognostic	40.4 ng mL^−1^	585 patients admitted to the ICU with severe sepsis or septic shock	47.6% 81.3%	[85]
bioactive adrenomedullin (Bio‐ADM)	22 min^[^ ^86^ ^]^	Diagnosis Sepsis	37 pg mL^−1^	1867 patients: 632 met sepsis‐3 criteria 267 had septic shock	61.0% 80.0%	[87]
		Prognostic	70 pg mL^−1^		42.0% 73.0%	[87]
Presepsin	4–6 h ^[^ ^88^, ^89^ ^]^	Diagnostic	median cut‐off: 600 pg mL^−1^	Pooled study: 18 studies 3470 patients	84.0% 76.0%	[90]
		Prognostic	350–1718 pg mL^−1^	Summary of 3 studies 693 patients	75.3% 50.8%	[91, 92, 93]

Pentraxin 3 (PTX3) is an acute phase protein that shows great potential as a prognostic biomarker. Studies have demonstrated a relationship between severity of sepsis and PTX3 concentration. High concentrations are also suspected to allow conclusions to be drawn about mortality.^[^ ^94^, ^95^, ^96^ ^]^ One biomarker with good potential for identifying bacterial infections is presepsin.^[^ ^93^, ^97^ ^]^ It shows significant potential for determining the severity of the infection and evaluating the efficacy of therapy.^[^ ^98^, ^99^, ^100^ ^]^ Additionally, the heparin‐binding protein (HBP) could be a suitable biomarker for distinguishing between bacterial sepsis and other types, as it is produced in response to bacterial infections.^[^ ^101^ ^]^ HBP has also shown great potential in the prediction of mortality.^[^ ^102^, ^103^ ^]^ Neutrophil cluster of differentiation 64 (nCD64) is another marker known for its ability to identify bacterial infections and differentiate between sepsis and no‐sepsis.^[^ ^78^, ^104^ ^]^

Other markers are useful to follow the response to therapy such as the soluble triggering receptor expressed on myeloid cells‐1 (sTREM‐1). This biomarker shows great promise in early diagnosis, the 28‐day mortality and, assessing disease severity.^[^ ^105^ ^]^ Dipeptidyl peptidase 3 (DPP3) is a biomarker which is released into the bloodstream due to cell death. It is considered to have the potential to predict organ failure and sepsis outcomes.^[^ ^85^, ^106^ ^]^ Another interesting biomarker is bioactive adrenomedullin (Bio‐ADM), which on the one hand gives an indication of the severity of the disease, as an increased concentration is often associated with more severe disease. Additionally, it is used as a predictor of organ failure and mortality.^[^ ^87^, ^107^, ^108^, ^109^ ^]^

This discussion shows that some markers provide similar information, while others complement each other. Therefore, next‐generation sepsis diagnostics should combine different biomarkers. The identification of a biomarker profile could also be used to differentiate subgroups of sepsis patients, which can be beneficial for treatment.^[^ ^110^, ^111^ ^]^ Another important aspect is monitoring the kinetics of biomarkers rather than measuring them at a single point in time. As previously mentioned, this approach can help to follow the course of the disease or the response to therapy.^[^ ^112^ ^]^

## 3 Conventional Diagnostic Tools

The gold standard for detecting a blood infection is to create a blood culture alone or followed by advanced analytical techniques such as mass spectrometry or polymerase chain reaction (PCR).^[^ ^113^ ^]^ Blood samples are taken from the patient and a cell medium is added in which the pathogens can multiply and be detected. Blood cultures require 2–5 days, which is slow compared to the dynamics of sepsis and causes delays in treatment decisions.^[^ ^19^, ^114^, ^115^, ^116^, ^117^ ^]^ In addition, the results of a blood culture can be influenced by the prior administration of antibiotics before sample collection. Furthermore, a risk of contamination exists during blood sampling, for example, due to improper disinfection, which can lead to invalid results. For more information we refer to Fabre et al., which provides a comprehensive summary of the use of blood cultures in diagnostics, focusing on the factors that influence the results of a blood culture.^[^ ^118^ ^]^

## 4 General Aspects of Biosensor Design

Biosensors are specifically designed to interact with biological materials and provide information about biological processes. They consist of three different components: a bio‐receptor which selectively captures the target, a transducer which converts the interaction into a measurable signal and a detector which measures this signal and translates it into a quantitative value such as a current.^[^ ^119^ ^]^

**Figure 2:**
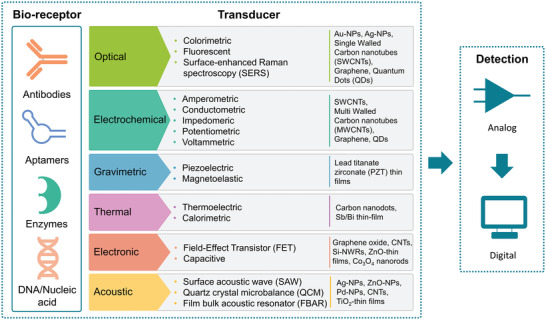
General design of biosensors with various detection and transduction components. The transducers are further divided into different categories with the appropriate nanomaterials in each category (optical,^[^ ^122^, ^123^, ^124^, ^125^, ^126^, ^127^, ^128^ ^]^ electrochemical,^[^ ^129^, ^130^, ^131^, ^132^ ^]^ gravimetric,^[^ ^120^, ^133^, ^134^ ^]^ thermal,^[^ ^135^, ^136^, ^137^, ^138^ ^]^ electronic,^[^ ^139^, ^140^, ^141^, ^142^, ^143^ ^]^ and acoustic^[^ ^144^, ^145^ ^]^).

The bio‐receptor is used to selectively bind the target substance and can be made e.g. from antibodies, enzymes, aptamers, or novel non‐conventional recognition motifs. This interaction leads to a cascade of actions, such as pH or temperature changes which can be detected by the transducer. The transducer converts these changes into an optical or electrical signal. The detector transforms the signal into a readable output. Biosensors can be characterized according to the transduction principle into electrochemical, optical, gravimetric, thermal, electronic, and acoustic biosensors (shown in **Figure** **2**).^[^ ^119^, ^120^, ^121^ ^]^

## 5 Nanomaterials as Transducer Elements for Optical Biosensors

Nanomaterials are structures with at least one dimension smaller than 100 nm. They are characterized by their large surface‐to‐volume ratio, which leads to increased reactivity and adsorption capacity.^[^ ^146^ ^]^ In addition to the altered reactivity, the electrical, magnetic, and optical properties of these materials also change.^[^ ^147^ ^]^ The change in optoelectronic properties in response to binding of analytes makes nanomaterials promising building blocks for biosensors. Here, we will focus on optical nanosensors because the optical readout has several advantages such as straightforward multiplexing by imaging arrays of sensors (see below).

Nanomaterials exhibit remarkable optical properties like metallic and semiconducting nanoparticles (NPs). The optical properties of metallic nanostructures can be explained by the oscillation of their delocalized conduction electrons when excited with light, a phenomenon called surface plasmon resonance (SPR). SPR can be further divided into surface propagating plasmons (SPP), which occur in metal films, and localized surface plasmons (LSPs) which are found in metal NPs.^[^ ^148^ ^]^ When metallic NPs are excited with electromagnetic radiation, a charge shift occurs in the NPs as the electron cloud oscillates relative to the nuclei, inducing an electric dipole aligned along the incident field. This creates a strong localized electromagnetic field on the surface of the NP.^[^ ^149^ ^]^ Two commonly used metal NPs are gold and silver NPs (AgNPs).^[^ ^150^ ^]^ Gold NPs (AuNPs) can change their color from red to blue depending on their size. In general, the size, shape, and composition of the NPs change their optical properties.^[^ ^151^, ^152^ ^]^

Semiconducting NPs, such as quantum dots (QDs), unlike metallic materials, have distinct valence and conduction bands separated by a band gap. When an electron is excited by light into the conduction band, a hole is created in the valence band forming an electron‐hole pair called an exciton. The relaxation of the electron back into the valence band can result in either radiative recombination, by emitting a photon, or non‐radiative recombination such as thermal decay. Reducing the size of a semiconductor to the nanoscale alters its optical properties due to the widening of the band gap between the conduction and the valence band, leading to a blue shift in the emission wavelength.^[^ ^153^, ^154^, ^155^, ^156^ ^]^

Optical biosensors can be categorized as either label‐free or label‐based. In a label‐free approach, the signal is produced directly by the interaction of the analyte with the transducer. In contrast, a label‐based approach generates the signal through the addition of a label that interacts with the analyte. This approach is used in colorimetric and luminescent methods.^[^ ^157^ ^]^ Optical biosensors can be further subdivided into colorimetric, fluorescence, and surface‐enhanced Raman spectroscopy (SERS) types (see Figure 2). Each type utilizes different nanomaterials suited for the specific application. The following section presents various nanomaterials as transducer elements in biosensors related to (single) sepsis targets.

### 5.1 Colorimetric Biosensors

Colorimetric biosensors detect color changes resulting from the binding of analytes, which can be observed with the naked eye or a simple optical detector. Various NPs show great potential for use in colorimetric biosensors, such as plasmonic NPs from gold or silver or doped liposomes.

#### 5.1.1 Metal Nanoparticles (NPs)

**Figure 3:**
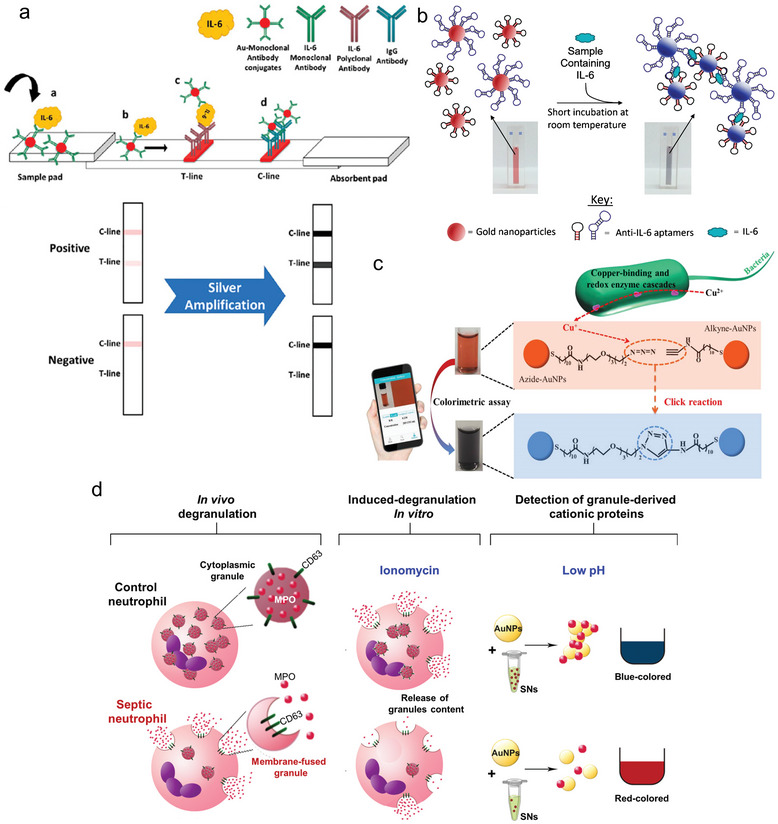
Lateral flow assays for inflammatory molecules based on colored NPs. a) Lateral flow approach for the colorimetric detection of IL‐6 using a lateral flow test, with an additional silver amplification step to increase the signal. Reprinted under the terms of the CC‐BY 4.0 license.^[^ ^160^ ^]^ (https://creativecommons.org/licenses/by/4.0/). Copyright 2021, Rahbar et al. b) Formation of aptamer‐mediated AuNP agglomeration by IL‐6 interactions in a sandwich structure with NPs. Reprinted with permission.^[^ ^163^ ^]^ Copyright 2019, Giorgi‐Coll et al.c) Illustration of the formation of covalent bonds between individual NPs due to the presence of bacteria and the associated color change, which is used for sensing. Reprinted with permission.^[^ ^164^ ^]^ Copyright 2019, American Chemical Society. d) Demonstration of the agglomeration‐based assay of AuNPs for the detection of sepsis‐induced hyperdegranulation. The agglomeration leads to a color change from red to blue. Reprinted with permission.^[^ ^165^ ^]^ Copyright 2021, American Chemical Society.

The localized surface plasmon resonance (LSPR) effect of Au‐NPs can be utilized in label‐based approaches, such as lateral flow tests.^[^ ^158^ ^]^ In these tests, AuNPs serve as labels, with the advantage of being visible to the naked eye. Au‐NPs are first conjugated with a recognition unit such as an antibody that is specific to the target. When the sample is applied to the test, a conjugate is formed between the modified NP and the target. The main component of the lateral flow test is a membrane with a test line, which consists of immobilized antibodies. The previously formed conjugate binds to the antibody on the test line, gradually coloring the line and making it visible.^[^ ^159^ ^]^ Besides being visible to the naked eye, AuNPs are also favored for their ease of preparation, though the sensitivity is relatively low. Rahber et al. developed a lateral flow test for the detection of IL‐6 with a limit of detection (LOD) of 5 pg mL^−1^ in serum. To overcome the low sensitivity, they added an AgNP‐based amplification step to enhance the signal (**Figure** **3**a).^[^ ^160^ ^]^ In another approach by Dong et al., amplification was achieved through an additional gold deposition step.^[^ ^161^ ^]^ Alba‐Patiño et al. demonstrated the ability of AuNPs to be scanned with a mobile phone. They developed a test for determining IL‐6 based on the previously mentioned sandwich structure between AuNPs and the target.^[^ ^162^ ^]^

In addition to visualization through enrichment at a single point in a test, plasmonic coupling caused by aggregation can also be used as a detection method.^[^ ^166^ ^]^ Here, the color of the NPs shifts from red to blue due to target‐mediated aggregation.^[^ ^167^ ^]^ Santopolo et al. developed a colorimetric biosensor based on the destabilization of the AuNP system through the release of the desired analyte (Figure 3d). In their work, they analyzed the degranulation status of septic cells. They synthesized citrate‐capped AuNPs to determine the amount of cationic proteins in the solution based on their state of agglomeration. For their experiment, they used myeloperoxidase (MPO) as an example protein. They demonstrated that the addition of MPO caused the AuNPs to agglomerate, resulting in a color change of the solution.^[^ ^165^ ^]^ As an alternative to destabilizing the NPs, the color change can also be induced by a reaction triggered by the analyte, causing the NPs to covalently bond together (Figure 3c). Mou et al.^[^ ^164^ ^]^ used this approach to detect bacteria based on a color change that can be seen by the naked eye or with a mobile device. They synthesized two different AuNPs species, one functionalized with an alkyne and the other with an azide group. The detection principle leverages the bacteria's ability to reduce Cu^2+^ to Cu^+^ to catalyze the alkyne‐azide cycloaddition. Therefore, the sample is pretreated with CuCl_2_. The Cu^+^ ion initiates the click reaction between the two types of AuNPs, which is then detected by a color change. Both approaches provide a color change that can already be identified with the naked eye. In a similar approach, Giorgi‐Coll et al.^[^ ^163^ ^]^ (Figure 3b) developed an assay exclusively for the detection of IL‐6 by the agglomeration of AuNPs functionalized with aptamers.

AgNPs similar to AuNPs are used because of their LSPR effect.^[^ ^168^ ^]^ Anh Thu et al. demonstrated a target‐mediated aggregation approach to detect *Staphylococcus aureus* by using this effect.^[^ ^169^ ^]^

Metallic NPs such as AuNPs and AgNPs are well‐suited for use in colorimetric biosensors due to their unique optical properties. Their visibility to the naked eye makes AuNPs particularly interesting for lateral flow experiments, where they become detectable by binding to the target molecule and accumulating at a specific site in the assay. Moreover, the ability of plasmonic NPs to exhibit colorimetric shifts through aggregation holds significant potential for colorimetric assays, where aggregation is induced by binding to the target.

#### 5.1.2 Dye‐Based Nanomaterials

However, also other nanomaterials offering great potential in colorimetric methods. In their work, Sun et al. presented a paper‐based device that can not only diagnose the presence of a bacterial infection, but also monitor drug resistance. To achieve this capability, they combined the two indicators bromothymol blue (BTB) and nitrocefin in a single test. BTB changes color from green to yellow in an acidic environment, which can occur during bacterial infections. Conversely, nitrocefin reacts with *β*‐lactamase produced by resistant bacteria, leading to a color change. They used *E. coli* as a test system.^[^ ^170^ ^]^ In another approach, Zhang et al. fabricated a nanofiber‐based colorimetric biosensor for *E. coli*. The nanofibers consist of poly(vinyl alcohol‐co‐ethylene) and were functionalized with 5‐bromo‐4‐chloro‐3‐indolyl‐*β*‐D‐glucuronide sodium salt trihydrate. The detection mechanism is based on the functionalized nanofibers changing color due to hydrolysis of the modification by the *β*‐glucuronidase secreted by *E. coli* bacteria. The color change allows to detect infections.^[^ ^171^ ^]^ Furthermore, the use of dye‐loaded liposomes is also possible. Rink et al. demonstrated a lateral flow approach for the detection of IL‐6 using liposomes loaded with sulforhodamine B. They functionalized the synthesized liposomes with the respective antibody and achieved an LOD of 1 pg mL^−1^ compared to the reference based on Au‐NPs and showed an LOD of 0.025 ng mL^−1^, which is therefore a good alternative to the conventionally used Au‐NPs.^[^ ^172^ ^]^

### 5.2 Fluorescent Biosensors

Compared to colorimetric biosensors, fluorescence‐based biosensors use changes in fluorescence induced by the target molecule. In contrast to colorimetric biosensors, fluorescence biosensor detects the emission of a signal. The advantage of fluorescence is their (typically) higher signal‐to‐noise ratio. As a result, fluorescence measurements are more sensitive and lower concentrations of a target substance can be detected. In multiplexing approaches, it can be advantageous that different fluorophores can be used for a single sample, which can be excited at different wavelengths. Therefore, several markers can be detected simultaneously in one sample, whereby the readout can be performed separately. Various readout methods can be distinguished, such as fluorescence intensity changes, lifetime, quantum yield, wavelength shifts, or anisotropy.^[^ ^173^, ^174^ ^]^ Numerous nanostructures have great potential in biosensing, for example carbon nanotubes (CNTs), graphene, up‐conversion NPs, or QDs.^[^ ^175^, ^176^, ^177^, ^178^, ^179^ ^]^ This method can also be performed label‐based or label‐free. In a fluorescent biosensor, a transducer is used that is itself fluorescent and changes its fluorescence properties when the analyte binds to it, or the analyte is tagged with a fluorescent label through the binding. The successful binding and the concentration in the sample can be determined from the fluorescence changes. The material properties determine the chemical design space and we will discuss a few important examples.

#### 5.2.1 Graphene Oxide‐Based Nanomaterials

**Figure 4:**
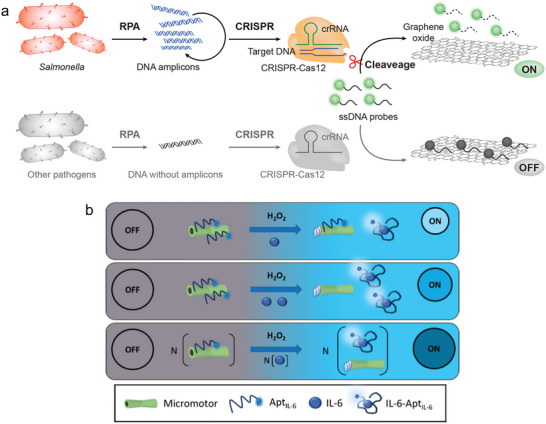
Graphene oxide‐based sensing approaches. a) Illustration of the GO‐CRISPR system, with a first amplification step, followed by the actual sensing step. Reprinted under the terms of the CC‐BY 4.0 license^[^ ^180^ ^]^ (https://creativecommons.org/licenses/by/4.0/). Copyright 2024, Kasputis et al. b) Visualization of the micromotor aptaassay and demonstration of the enhanced response by increasing the target concentration. Reprinted under the terms of the CC‐BY 4.0 license^[^ ^181^ ^]^ (https://creativecommons.org/licenses/by/4.0/). Copyright 2024, Gordón et al.

Graphene and related materials consist of a single layer of carbon atoms arranged in a hexagonal lattice.^[^ ^182^ ^]^ Kasputis et al. demonstrated the development of a graphene oxide CRISPR‐Cas12a (GO‐CRISPR) biosensor for the detection of *Salmonella typhimurium*. The sensing response is achieved through the fluorescence resonance energy transfer (FRET) effect. The process initially involves an amplification step of Salmonella DNA, followed by the addition of crRNAs, Cas12a proteins, and ssDNA‐FAM probes. The presence of the target activates the CRISPR system, which causes cleavage of the ssDNA FAM probes, resulting in shorter fragments. In the next step, graphene oxide is added to the mixture. As only longer ssDNA is able to interact with the graphene oxide by π−π stacking, the shortened ssDNAs are not able to bind. The binding through graphene oxide leads to the quenching of the fluorescence signal through the FRET effect. If the sample does not contain Salmonella, the fluorescence decreases (**Figure** **4**a).^[^ ^180^ ^]^ This system can be applied to different nanomaterials as shown by Cheng et al.^[^ ^183^ ^]^ They compared the ability of graphene oxide versus AuNPs for quenching and enhancement strategies to detect DNA.

However, other nanomaterials are also produced from graphene oxide, such as the micromotors developed by Gordón et al. These micromotors are fabricated using a template‐based electrodeposition process consisting of an outer layer made of graphene oxide, an intermediate layer of nickel and an inner layer from platinum. They are bubble‐propelled and move continuously through the sample. The movement is achieved through a catalytical reaction with H_2_O_2_. To develop a sensor for IL‐6, the micromotors were modified with aptamers selective for IL‐6. The aptamers contain a fluorophore that is quenched when bound to the micromotors. If the sensor is now applied to a sample containing IL‐6, the aptamers detach and the fluorescence is no longer quenched. Consequently, the fluorescence increases in proportion to the IL‐6 concentration in the sample (Figure 4b).^[^ ^181^ ^]^

#### 5.2.2 Carbon Nanotubes (CNTs)

CNTs consist of rolled‐up graphene sheets and are divided into multi‐walled (MWCNTs) and single‐walled (SWCNTs) CNTs. They are of great interest for fluorescent biosensing applications due to their high photobleaching resistance, various functionalization options, low synthesis costs, and multicolor emission by different chiralities. Semiconducting SWCNTs emit fluorescence signals in the near‐infrared (NIR) (870–2400 nm).^[^ ^175^ ^]^ Their fluorescence properties are sensitive to their chemical environment, making them suitable for biosensing applications. SWCNTs can be modified with different ligands to bind specific targets, which changes the fluorescence properties such as intensity or emission peak wavelength.

**Figure 5:**
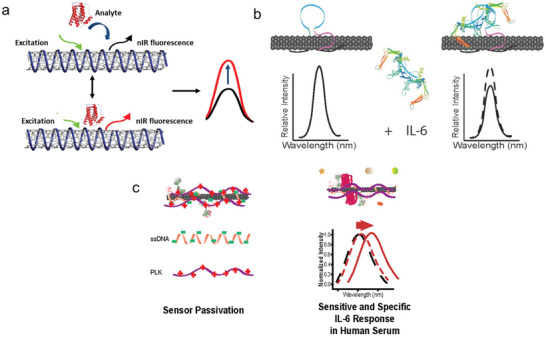
SWCNT‐based biosensors for the detection of IL‐6. a) Representation of the functionalization of the polymer‐functionalized SWCNTs, which interact with IL‐6 resulting in an increase of the fluorescence signal. Reprinted with permission.^[^ ^184^ ^]^ Copyright 2023, American Chemical Society. b) Demonstration of the interaction of an aptamer functionalized SWCNTs and the corresponding change in intensity. Reprinted under the terms of the CC‐BY 4.0 license^[^ ^185^ ^]^ (https://creativecommons.org/licenses/by/4.0/). Copyright 2024, Ryan et al. c) Illustration of SWCNTs functionalized with ssDNA modified with the IL‐6‐antibody and with Poly‐L‐Lysine for passivation and the corresponding wavelength shift by binding of IL‐6. Reprinted under the terms of the CC‐BY 4.0^[^ ^186^ ^]^ (https://creativecommons.org/licenses/by/4.0/). Copyright 2024, Gaikwad et al.

The following presents various modification strategies for the detection of IL‐6. Jin et al. demonstrated a corona phase molecular recognition (CoPhMoRe) approach. They modified SWCNTs with a library of different ligands and tested which of them reacted specifically to IL‐6. By screening the modified SWCNTs against various targets, they identified a polymer‐based recognition unit that can detect IL‐6 (**Figure** **5**a).^[^ ^184^ ^]^ In comparison, Ryan et al. functionalized SWCNTs with an aptamer identified to be specific for IL‐6 by the systematic evolution of ligands by exponential enrichment (SELEX) process. The aptamer is non‐covalently bound to the surface of the SWCNTs. They demonstrated an LOD of 105 ng mL^−1^ (Figure 5b).^[^ ^185^ ^]^ In contrast, Gaiwad et al. used an antibody‐based approach. They first modified SWCNTs with an amine‐modified ssDNA (5′‐(TAT)6)/3AmMO/‐3′), which was then used to bind a monoclonal IL‐6 antibody via carbodiimide coupling. To prevent unspecific interactions with serum, the surface was passivated with different agents, like bovine serum albumin (BSA), Poly‐L‐Lysine and casein (Figure 5c).^[^ ^186^ ^]^ SWCNTs can also be modified covalently through quantum defects (sp^3^ defects). Kim et al. demonstrated an approach for developing a covalently bound recognition unit based on a supercharged single‐chain antibody fragment (scFv).^[^ ^187^ ^]^ To synthesize the sensors, a 4‐carboxyl aryl defect was introduced in a first step by diazonium chemistry. These defects were then used to covalently bind the scFv by carbodiimide coupling. The resulting sensor is based on the ligand‐induced folding of scFv by IL‐6, which leads to a solvatochromic shift of the fluorescence signal.

These sp^3^ defects can be further modified with bio‐recognition units or used to directly bind the target molecules, as shown by Ma et al. In general, sp^3^ defects can change the optical properties of SWCNTs and lead for example to a brightening of the fluorescence signal.^[^ ^188^ ^]^ Molecules such as H_2_O_2_ can also be detected by specific modification of SWCNTs. Wu et al. demonstrated a sensor for H_2_O_2_ based on the modification of SWCNTs with a hemin aptamer, which binds to hemin that reacts with H_2_O_2_ to form hydroxyl radicals. SWCNTs are known to react with reactive oxygen species (ROS) by quenching the fluorescence signals. This effect can then be used to draw conclusions about the H_2_O_2_ concentration.^[^ ^189^ ^]^

#### 5.2.3 Quantum Dots (QDs)

**Figure 6:**
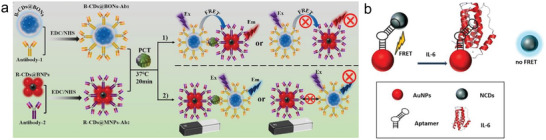
Detection of inflammatory molecules through QD‐based biosensors. a) Visualization of the preparation mechanism of the different NPs for the detection of PCT and the respective sensor model. Reprinted with permission.^[^ ^190^ ^]^ Copyright 2023, Elsevier. b) Schematic representation of the FRET‐based sensing mechanism for the detection of IL‐6 based on gold and carbon QDs (NCDs). Reprinted with permission.^[^ ^191^ ^]^ Copyright 2024, Springer Nature

QDs are another class of fluorescent nanomaterials. They have been optimized to be very bright and therefore they are typically less sensitive to their environment as for example SWCNTs. Zhou et al. developed an immunochromatographic assay for the detection of PCT based on quantum dot nanobeads with an LOD of 0.0625 ng mL^−1^. They embedded oleylamine‐coated CdSe/ZnS into a polymer matrix to obtain quantum beads (OB). As a result, they achieved a ≈900‐fold stronger fluorescence signal compared to a single QD. The resulting QBs were then further functionalized with PCT antibodies. The accuracy of the test was validated in serum samples.^[^ ^192^ ^]^ In addition to labeling, a label‐free, FRET‐based sensor approach is also possible. To this end, Mahani et al. developed a test based on nitrogen‐doped carbon QDs (NCDs) and Au‐NPs for the detection of IL‐6. First, they synthesized fluorescent NCDs and AuNPs functionalized with an aptamer. The sensing is based on the quenching of the NCDs fluorescence signal through the AuNps. The aptamer is able to bind the NCDs. In the presence of IL‐6 in the sample, the configuration of the aptamer changes, releasing the NCDs and thus the fluorescence is no longer quenched. This concentration‐related fluorescence recovery enables the determination of the IL‐6 concentration in the sample (**Figure** 6b).^[^ ^191^ ^]^ Chen et al. showed a similar approach for the detection of CRP, in which the fluorescence properties of CdSe/ZnS QDs were amplified through the presence of silica coated Au‐NPs.^[^ ^193^ ^]^ To amplify the signal, the prior concentration of the sample can be useful. Liu et al. demonstrated an immunomagnetic approach for the detection of PCT, in which magnetic NPs were used to extract the analyte to be detected from the sample and then perform the measurement in an enriched solution. For comparison, they demonstrated sensor performance with the same materials without the extraction and concentration step in a FRET‐based immunosensor (Figure 6a).^[^ ^190^ ^]^

### 5.3 Surface‐Enhanced Raman Spectroscopy (SERS)

Raman spectroscopy identifies molecules by analyzing vibrational modes. A sample is irradiated with monochromatic light, and the scattered light is then analyzed. Raman signals are specific to certain molecules due to their specific vibrational and rotational modes. However, the process has a low efficiency.^[^ ^194^ ^]^ Raman signals can be amplified e.g. by either improving the polarizability of the molecule or amplifying the external electric field.

SERS addresses this issue by enhancing the signal using nanomaterials such as gold or AgNPs. Signal amplification in SERS can occur through two effects: chemical or electromagnetic enhancement. Chemical enhancement is limited to molecules in direct contact with the nanostructure surface and is based on a charge–transfer complex. This effect influences the molecule's polarizability.^[^ ^194^, ^195^ ^]^In contrast, electromagnetic amplification, amplifies the external electric field acting on the molecule.^[^ ^194^ ^]^ This effect can be further enhanced by using dimers that form hot spots. Molecules in the vicinity of these hotspots exhibit drastically increased Raman signals, enabling the detection of single molecules.^[^ ^196^, ^197^, ^198^ ^]^ Data show that the SERS effect depends on the distance between the molecule and the NP. As the distance between the molecule and the NP increases, the electromagnetic field at the particle's surface decreases, reducing the field's influence.^[^ ^199^ ^]^

**Figure 7:**
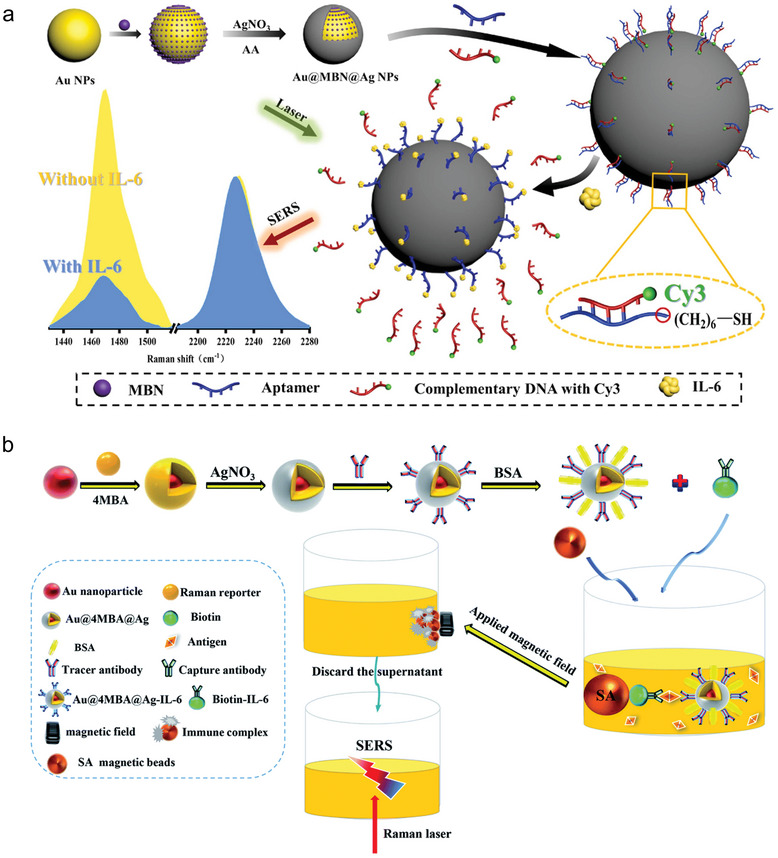
SERS‐based biosensors for the detection of inflammatory molecules. a) Illustration of the self‐calibrating aptamer‐based nanosensor for the detection of IL‐6. The spectra in the lower part on the left side show the Raman signal depending on the presence of IL‐6 and on the right side the reference acting as calibration. Reprinted with permission.^[^ ^200^ ^]^ Copyright 2023, Elsevier. b) Demonstration of the SERS magnetic immunoassay for the detection of PCT. The first part shows the synthesis of the nanosensor and in lower part the test in serum. Reprinted with permission^[^ ^201^ ^]^ (https://doi.org/10.1039/D0AY02304C). Copyright 2021, The Royal Society of Chemistry.

Li et al. demonstrated a sensor for PCT based on Bi_2_WO_6_‐GO and 4‐(2‐(3‐(dicyanomethylene)‐5,5‐dimethylcyclohex‐1‐en)vinyl)phenyl)boronic acid (BP) with an LOD of 0.31 pg mL^−1^ and a detection time <8 min. Their approach relies on chemical mechanism‐based SERS. The sensor experiment was performed in whole blood.^[^ ^202^ ^]^ In comparison, Selimoğlu et al. created a biosensor for PCT with an LOD of 4 ng mL^−1^ by developing single‐layer graphene doped with AgNPs modified with antibodies.^[^ ^203^ ^]^ An aptamer self‐calibration sensor for the detection of IL‐6 was presented by Huang et al. They synthesized a core–shell structure consisting from a gold core with two different shells. The inner shell was made of 4‐mercaptobenzonitrile (MBN) and the outer shell of silver. This arrangement not only enhances, but also allows MBN to function as an internal standard molecule signal at the biological Raman silent region 2227 cm^− 1^. The particle was further functionalized with an aptamer which was then hybridized with a complementary strand containing a Raman signal molecule, cyanine 3 (Cy3). In the presence of IL‐6, the higher affinity of the aptamer causes the Cy3 containing strand to be released. Consequently, the SERS signal of the Cy3 decreases. The decrease in Cy3 is compared to MBN to create a self‐calibrating aptamer sensor. The LOD of the sensor was 0.056 pg mL^−1^ (**Figure** **7**a).^[^ ^200^ ^]^ Wang et al. also demonstrated a NP‐based approach to detect IL‐6 using an antibody–antigen–antibody sandwich structure. They synthesized a core shell structure with a gold core, an inner shell of the Raman receptor 4‐mercaptobenzoic acid (4MBA), and an outer silver shell to enhance the Raman signal. Here, antibodies were used as a recognition unit. After coupling the particles to IL‐6, the conjugate was further coupled to magnetic beads. With the help of an external magnetic field the conjugate could then be extracted and redispersed in another medium. Subsequently, the Raman signal of the solution could be detected to determine IL‐6 (Figure 7b).^[^ ^201^ ^]^ Even though Raman‐based approaches provide a lot of chemical information typically SERS and recognition units are necessary as discussed above for detection in complex matrices.

## 6 Multiplexed Nanosensor‐Based Strategies for Point‐of‐Care (POC) Applications

### 6.1 General Design Considerations

**Figure 8:**
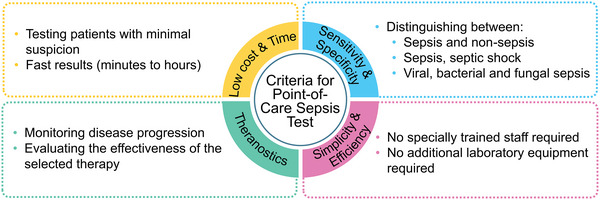
Requirements for the ideal point‐of‐care (POC) biosensor.

Numerous nanosensors have been developed to recognize single sepsis‐related targets as discussed in the previous section. However, sepsis diagnosis should be performed at POC, as sepsis is a time‐dependent disease. An ideal POC biosensor should be cost‐efficient and require minimal infrastructure (**Figure** **8**). This approach would enable testing of patients at the slightest suspicion of sepsis and verification of therapeutic success through continuous testing (theranostics). Rapid identification of the cause of sepsis (bacterial, viral, or fungal) is crucial for effective treatment in order to enable targeted treatment and avoid unnecessary antibiotics (rapid turnaround time). Since the success of treating a septic patient depends on the time required to administer the correct therapy, time is the critical factor that should be prioritized in the development of a new test procedure. Additionally, the POC device should be simple to use, allowing untrained personnel to operate it effectively.

Sensitivity and specificity are two other properties that play an important role in biosensors. Understanding the difference between the diagnostic and analytical definitions of these terms is essential. In diagnostics, sensitivity refers to the ability of the test, to correctly identify patients with a disease. The specificity of a test indicates how reliably it classifies healthy patients as negative.^[^ ^204^ ^]^ In analytical terms, sensitivity refers to a test's ability to detect small concentration and small changes in the concentration of a given target. This primarily is determined by its responsiveness and its LOD. A distinction is also made between selectivity and specificity in analytical contexts. Specificity means that a test is specific for one analyte without being affected by other molecules. Selectivity describes a test's ability to distinguish between the target substance and other substances in the sample.^[^ ^205^ ^]^ The combination of various tests of different types in a single array‐based sensor system is important as it can create synergies.^[^ ^206^ ^]^ Overall, a single marker is unlikely to be sufficient enough to clearly diagnose sepsis, its origin, severity, etc. Therefore, we believe that multiplexing strategies are the key to solve this challenge, and we will discuss recent advances.

To further refine the criteria for effective POC diagnostics, the REASSURED criteria developed by Land et al. have emerged as comprehensive framework.^[^ ^207^ ^]^ These criteria are designed to ensure that POC diagnostic tools are not only scientifically robust, but also practical and accessible, particularly in low‐resource settings. The REASSURED approach builds on the previous ASSURED criteria by incorporating elements such as connectivity and ease of use to enhance the implementation and utility of diagnostic tests in real‐world conditions.

In the context of sepsis, early diagnosis and treatment are critical for improving patient outcomes and the REASSURED criteria provide valuable guidance for developing effective POC diagnostic tools. Sepsis POC tests must be designed to integrate with electronic health records (EHR) or telemedicine platforms, ensuring that critical patient data is immediately accessible for clinical decision‐making (**R**eal‐time connectivity). Additionally, the collection of specimens should be as simple and non‐invasive as possible, employing techniques like finger‐prick blood samples to reduce patient discomfort and streamline the process (**E**ase of specimen collection).

Affordability is another crucial factor, especially in resource‐limited settings, as lower costs will enable broader access to these life‐saving diagnostics (**A**ffordable). High sensitivity is vital to detect even low levels of sepsis biomarkers early in the disease's progression, ensuring timely intervention (**S**ensitive). Moreover, the specificity of the test must be high to accurately distinguish sepsis from other inflammatory conditions, thereby minimizing false positives and unnecessary treatments (**S**pecific).

To be truly effective, these tests must also be user‐friendly, with clear instructions and minimal steps required to obtain results, which is particularly important in settings with varying levels of healthcare expertise (**U**ser‐friendly). The rapid nature of sepsis means that every minute counts. Thus, the test must deliver results quickly, ideally within an hour, and function reliably under different environmental conditions (**R**apid and robust). Finally, the tests should be self‐contained and portable, ensuring they can be used in remote or resource‐limited environments without the need for specialized equipment (**E**quipment‐free) and distributed effectively to reach all potential users (**D**eliverable to end‐users). By meeting these criteria, POC diagnostics for sepsis can significantly improve early detection and treatment, ultimately saving lives and alleviating the strain on healthcare systems globally.

As we explore the current and emerging POC technologies, we focus on those approaches that best align with the REASSURED criteria. Technologies such as lateral flow tests, microfluidic devices, multiplexing platforms, and advanced biosensors are particularly relevant because they exemplify key principles like affordability, rapid result delivery, and ease of use, which are critical for effective sepsis diagnosis and treatment. For instance, LFTs are not only cost‐effective and straightforward to use, but they also offer quick results, making them ideal for early sepsis detection. Similarly, microfluidic devices are being engineered to quickly process small blood samples, ensuring that they meet the needs for portability and user‐friendliness. Multiplexing platforms, which can simultaneously analyze multiple biomarkers, provide the high sensitivity and specificity required for accurate sepsis diagnostics. Emerging biosensors, which can simultaneously detect multiple sepsis biomarkers, provide the high sensitivity and specificity required for accurate diagnostics and are increasingly developed to be both equipment‐free and suitable for remote locations. Continued innovation in these areas is essential to address the global challenge of sepsis effectively.

### 6.2 Multiplexing Platforms for Optical POC Strategies

An ideal POC biosensor could be used without access to a specialized laboratory. However, the right platform is required. For example, the previously presented Au‐NPs, which rely on agglomeration or destabilization, are unsuitable for surface‐bound sensor technology, such as in a lateral flow approach because a liquid phase is required. Therefore, this factor must be considered when selecting the appropriate technology for the test. As mentioned above, sepsis involves various processes associated with different biomarkers. A biomarker‐based test must consider this complexity and combining different markers (multiplexing) is assumed to maximize the information content. The following section presents different technologies that could serve as platforms for POC sepsis tests.

#### 6.2.1 Nano‐Microarrays

A nano‐ or microarray is a sensing platform with various spots that perform different analytical tasks simultaneously.

**Figure 9:**
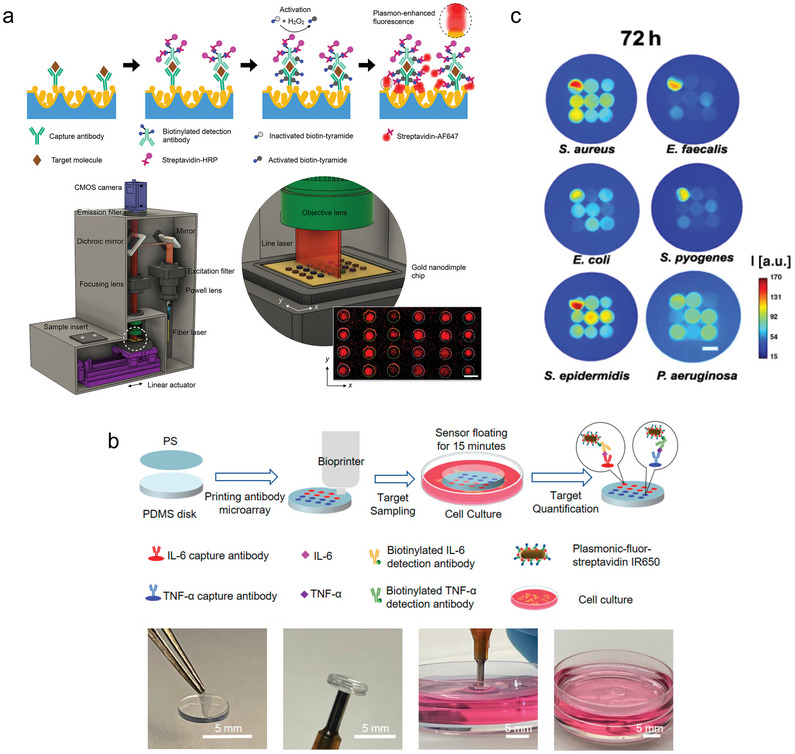
Nano‐microarray technologies for simultaneous detection of pathogens and related molecules. a) The upper part illustrates the sensor and its interaction with the analyte, including the enhancement step. The lower part displays the sensor and the screening setup. Reprinted with permission.^[^ ^208^ ^]^ Copyright 2023, American Chemical Society. b) Schematic and optical images depicting the development of the sensor array and the execution of the test. Reprinted under the terms of the CC‐BY 4.0 license^[^ ^209^ ^]^ (https://creativecommons.org/licenses/by/4.0/). Copyright 2023, American Chemical Society. c) Image of a fluorescent sensor array consisting of various SWCNT‐based sensors embedded in a hydrogel matrix, which fingerprints bacteria. Reprinted under the terms of the CC‐BY 4.0 license^[^ ^210^ ^]^ (https://creativecommons.org/licenses/by/4.0/). Copyright 2020, Nißler et al.

Chin et al. developed a chemifluorescence assay based on multiplexed detection of IL‐3, IL‐6, IL‐10, IL‐1β, TNF‐α, and MCP‐1 within 1 h, achieving sub‐pg mL^−1^ sensitivity. They validated the accuracy of their test with 20 clinical plasma samples. The test is based on DuPLUS (dual‐enhanced plasmonic ultrasensitive sensing), combining two signal enhancement strategies: localized plasmonic coupling and tyramide chemifluorescence signal amplification. They created a chip based on AuNPs on a gold nanodimple arrays. Various antibodies specific for the respective target molecules were used as recognition units. To further enhance the signal, they used tyramide chemifluorescence signal amplification. Binding the AF647 fluorophore to the assay significantly enhanced the fluorescence emission (**Figure** **9**a).^[^ ^208^ ^]^ Along the same lines, Guo et al. presented a buoyant (i.e., floating) sensor for detecting cytokines in culture media (Figure 9b). They created a sensor patch that floats on the surface of the culture medium. They prepared a sensor batch from a poly(dimethylsiloxane) (PDMS) disk with a flexible polystyrene film on top. The respective antibodies were printed on the film in an array structure. This array was then placed into the cell media and after 15 min the patch was removed from the liquid and treated first with detection antibodies, then with a plasmon‐enhanced fluorescence‐linked immunosorbent assay (pFLISA). Therefore Silver‐coated gold nanorods were functionalized with a ≈3 nm thick siloxane copolymer layer, BSA‐conjugated IR650, and streptavidin (so‐called Plasmonic Fluors (PFs)), and were then applied to the patch. At this stage, this technology is not suitable for a POC strategy, but with further optimization, it shows great potential for diagnostics outside of cell cultures.^[^ ^209^ ^]^ Another approach is to detect directly bacterial motifs. Nißler et al. developed a test based on nine different NIR fluorescent SWCNT‐based sensors to detect the bacteria *S. aureus*, *S. epidermidis*, *S. pyogenes*, *E. faecalis*, *E. coli*, and *P. aeruginosa*. Detection is based on metabolites secreted by these bacteria. By combining nine different sensors in a hydrogel array, a single array was developed. They all exhibit fluorescence properties in the NIR range. By incubating them with the respective bacterial suspension the individual sensors reacted differentially, which allowed to fingerprint the bacteria. They also demonstrated the effectiveness of their test in human synovial fluid, showing no impairment in this realistic matrix (Figure 9c).^[^ ^210^ ^]^ This result shows that fingerprinting of bacteria is possible by using multiple sensors.

#### 6.2.2 Fluorescence‐Linked Immunosorbent Assay (FLISA)

**Figure 10:**
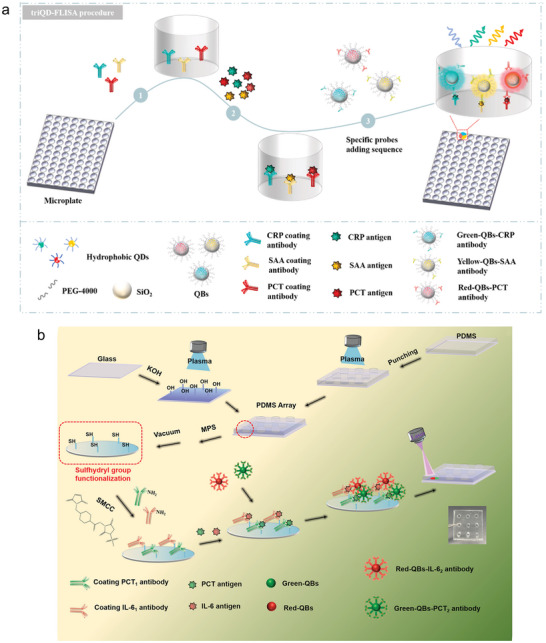
FLISA assays for the simultaneous detection of multiple inflammatory molecules. a) Illustration of the different processing steps to develop a three‐color QB‐based FLISA for the simultaneous detection of SAA, PCT, and CRP. First, a microplate is modified with the respective antibody, then the target molecule is applied, and three different QDs are added. Each of the QDs is modified with an antibody to specifically bind one of the targets. Adapted with permission.^[^ ^213^ ^]^ Copyright 2023, Elsevier. b) Schematic representation of the fabrication of the PDMS‐based immunosensor for the detection of PCT and IL‐6. Demonstration of the antigen coupling step, the reaction with the target molecule, and the subsequent reaction with the corresponding QBDs to identify the different analytes. Reprinted with permission.^[^ ^214^ ^]^ Copyright 2024, American Chemical Society.

A fluorescence‐linked immunosorbent assay (FLISA) is a test in which fluorophores are immobilized for detection. As shown in **Figure** **10**a, a microtiter plate is first modified with antibodies or antigens. A blocking step can then be performed to prevent unspecific interactions. In the next step, the plate is incubated with the sample to be analyzed. A washing step removes unbound substances and then a fluorophore is added to detect the target.^[^ ^211^, ^212^ ^]^ Lv et al. developed a three‐color quantum bead (QB)‐based fluorescence‐linked immunosorbent assays (tQBs‐FLISA) for the detection of CRP, serum amyloid A (SAA), and PCT. In their work they used CdSe/ZnS core/shell QDs with different optical properties (PL = 516 nm, PL = 580 nm, and PL = 630 nm) to identify the different targets. First, they synthesized three different CdSe/ZnS core/shell QDs. To amplify the fluorescence signal, the obtained QDs were used to develop QBs in a second step. These QBs were then modified with the respective antibodies for the different targets to obtain yellow‐QBs‐SAA antibody, green‐QBs‐CRP antibody, and red‐QBs‐PCT antibody probes. They were able to detect the different targets simultaneously with an LOD of 0.48 ng mL^−1^ for CRP, 0.42 ng mL^−1^ for SAA, and 10 pg mL^−1^ for PCT. The effectiveness of the test was also demonstrated in human negative serum (Figure 10a).^[^ ^213^ ^]^

Zhao et al. also introduced a FLISA‐based sensor to detect IL‐6 and PCT. With their approach they achieved an LOD of 24 pg mL^−1^ for IL‐6 and 32 pg mL^−1^ for PCT. In their work, they first optimized the surface functionalization of the sensor to ensure the proper orientation of the immobilized antibodies and to prevent non‐specific adsorption and interaction of the QDs with the surface. They created a PDMS array functionalized with different antibodies. To enhance the fluorescence signal they used QBs instead of QDs in this work. Another advantage of QBs is their increased surface area, which allows them to bind more biomolecules. In this work, they synthesized QBs emitting different light, Green‐QBs (PL = 517 nm) and Red‐QBs (PL =  20 nm), to differentiate between the different targets. The combination of both QBs enabled the simultaneous detection IL‐6 and PCT (Figure 10b).^[^ ^214^ ^]^

#### 6.2.3 Microchannel/Microfluidic Systems (Lab on Chip)

Microchannel or microfluidic systems, also known as lab‐on‐a‐chip devices, are small sensing platforms that contain microchannels capable of transporting small volumes of a sample into analysis chambers or reaction sites through capillary forces. The channels can be modified in various ways, and different channels can run side by side, with each representing either a replicate or an individual test series.

**Figure 11:**
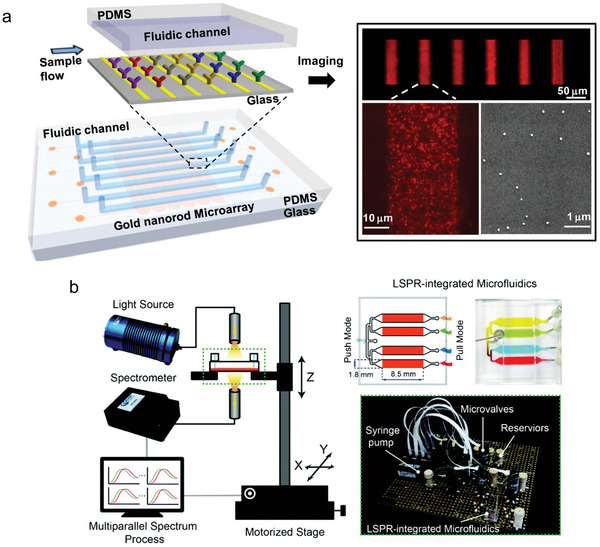
Microfluidic systems for simultaneous detection of sepsis‐associated targets. a) Schematic representation of the microfluidics used for testing IL‐2, IL‐4, IL‐6, IL‐10, IFN‐γ, and TNF‐α. Each channel contains nanorods that were previously deposited on a glass substrate and functionalized with specific antibodies. On the right side, images of the microarray captured under dark field microscopy and scanning electron microscopy (SEM) are also presented. Reprinted with permission.^[^ ^215^ ^]^ Copyright 2015, American Chemical Society. b) Schematic and optical images of the detection unit and the LSPR microfluidics. Reprinted with permission.^[^ ^216^ ^]^ (https://doi.org/10.1039/D0AN01201G). Copyright 2020, The Royal Society of Chemistry.

Chen et al. presented a label free detection method based on LSPR method. They developed a multiplex approach to simultaneously detect different cytokines (interleukin‐2 (IL‐2), interleukin‐4 (IL‐4), IL‐6, interleukin‐10 (IL‐10), interferon‐gamma (IFN‐γ), and tumor‐necrosis‐factor alpha (TNF‐α). Their sensor is based on gold nanorods that were functionalized with specific antibodies for each target cytokine. They created a chip that contains eight channels, six of which are functionalized with nanorods coated with different antibodies. The test requires an incubation time of 30 min and a sample size of only 1 µL, achieving a sensitivity of 5–20 pg mL^−1^, depending on the target cytokine. To validate the effectiveness of their test, they correlated the results of their LSPR assay with those from singleplex ELISA measurements. They used serum sample from healthy donors and added the different cytokines for comparison (**Figure** **11**a).^[^ ^215^ ^]^ Chen et al. demonstrated a four‐channel microfluidic system capable of detecting IgG, TNF‐α, IL‐6, and CRP within a testing time of 3.5 h and a sample volume requirement of 60 µL. The detection principle relies on the continuous measurement of the absorption spectra. When the analyte passes through the sensor, it can be detected by a shift in the wavelength. This label‐free semi‐quantitative multiplex approach has thus far only been tested in a buffer system and still requires validation through testing in real matrices (Figure 11b).^[^ ^216^ ^]^

#### 6.2.4 Paper‐Based Methods

Paper‐based methods can be categorized into different platforms: dipstick assays, lateral flow assays, and microfluidic paper‐based analytical devices (µPADs). Lateral flow tests were described in Section 5.1.1. A dipstick test also consists of a test strip with specific spots that react to a particular target. The test strip is immersed in the sample and then analyzed. Direct readouts can be obtained through color changes or by measuring with a suitable device. Dipstick test are primarily used for urine analysis. µPADs operate based on capillary effects. The sample is applied to a designated spot on the paper. From here, it is transported via capillary forces to the different sensing spots. Detection can also be based on color changes or analyzed with an additional device.^[^ ^217^ ^]^

**Figure 12:**
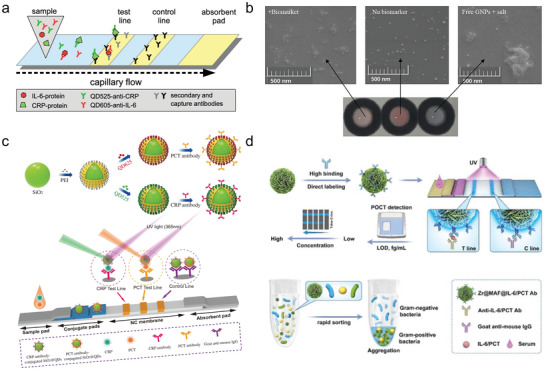
Paper‐based methods for multiplexed detection of sepsis related targets. a) Schematic of a lateral flow sandwich immunoassay for the simultaneous detection of IL‐6 and CRP. Two different QDs are used to identify the respective targets. Reprinted under the terms of the CC‐BY 4.0 license^[^ ^176^ ^]^ (https://creativecommons.org/licenses/by/4.0/). Copyright 2020, Ruppert et al. b) Demonstration of the sensor mechanism of the fabricated µPAD chip for detecting CRP and IL‐6. This illustration shows that the color of the test changes from red to blue in the presence of the analyte. Reprinted with permission.^[^ ^220^ ^]^ Copyright 2023, Elsevier. c) Synthesis of QDs‐coated silica NPs. Two different QDs were utilized to prepare a NP‐based assay capable of simultaneously detecting multiple targets within a single sample. To achieve this, each QD is conjugated to an antibody specific for its respective target. Reprinted with permission.^[^ ^218^ ^]^ Copyright 2020, Springer Nature. d) Demonstration of the MAF‐based LFAs used for the detection of IL‐6 and PCT, as well as the ability of the MAFs to differentiate between gram‐positive and gram‐negative bacteria. Reprinted with permission.^[^ ^221^ ^]^ Copyright 2023, American Chemical Society.

Ruppert et al. created a quantum dot‐labeled lateral flow assay for the simultaneous detection of IL‐6 and CRP (**Figure** **12**a). They utilized the ability of QDs to emit light at different wavelengths. These CdSe‐QDs were modified with antibodies specific to either CRP or IL‐6.^[^ ^176^ ^]^ In a similar approach, Yang et al. demonstrated a test based on a core‐shell structure of CdSe/ZnS‐COOH (excitation/emission 365/625 nm) and green CdSe/ZnS‐COOH (excitation/emission 365/525 nm) QDs for the detection of CRP and PCT. They first synthesized silica NPs as a core material, functionalized them with polyethyleneimine, and subsequently absorbed the different QDs (Figure 12c). In the final step, these NP systems were functionalized with the respective antibodies to develop two distinct sensors that emit different signals. The test is conducted as a standard lateral flow assay. Silica nanocarriers were chosen for their homogenous structure, high stability and good dispersibility.^[^ ^218^ ^]^ One year prior, the same group published another paper in which they used Fe_3_O_4_ magnetic NPs instead of silica cores. This change allows the nanosensors to first enrich targets in a given sample, which can then be extracted using a magnet and subsequently applied to the lateral flow test.^[^ ^219^ ^]^

In addition to NPs, also metal‐organic frameworks (MOFs) can be utilized for LFAs. Chai and colleagues developed an LFA capable of detecting PCT, IL‐6, and pathogenic bacteria simultaneously (Figure 12d). The organic linker used in this study is an aggregation‐induced emission luminogen (AIEgen), resulting in the MOFs being referred to as MAFs. To detect the different targets, they functionalized their MAFs with the appropriate antibodies and then conducted a lateral flow test. They demonstrated that their MAFs‐based system is 100 times more sensitive than QDs. Moreover, they showed that MAFs can distinguish between gram‐positive and gram‐negative bacteria. However, the differentiation between the bacteria was performed in a separate experiment in solution, not within the lateral flow test. In summary, the MAFs demonstrate significant potential for use in POC applications.^[^ ^221^ ^]^

In addition to the lateral flow tests, microfluidic paper‐based analyzers (µPADs) are also available. Malekmohamadi et al. demonstrated a µPAD‐based colorimetric sensor approach for the detection of IL‐6 and CRP with an LOD of 0.05 ng L^−1^ and 0.43 mg L^−1^, respectively (Figure 12b). The detection principle is based on the aggregation of AuNPs. In this method, the NPs are first modified with the respective aptamers. In the presence of the target molecule, the aptamer detaches from the NP, and the addition of salt induces the aggregation of the NP, resulting in a color change from red to blue. The color change allows for the measurement of IL‐6 and CRP concentrations.^[^ ^220^ ^]^ All these examples show that simple paper‐based assays are within reach, but they have to be tested under the most realistic conditions.

## 7 Outlook and Perspectives

Sepsis is still one of the major problems in medicine with a huge burden for the healthcare system. Even though sepsis has been known for hundreds of years no sufficient molecular tool exists yet to clearly identify it. Constant efforts have been made for many years to reduce the sepsis burden, but no diagnostic tool has yet been developed that could replace the slow standard (blood culture). In this review article, we discussed important biomarkers and nanomaterial‐based sensors for sepsis‐related molecules. These examples clearly show that there has been tremendous progress. Developing the ideal biosensor requires the creation of a platform capable of detecting various types of biomarkers, such as inflammatory molecules, DNA, and proteins. In other words, the use of multiplexing approaches is crucial, as no single target for sepsis currently exists and will most‐likely never be found. Overall, the major bottleneck in advancing diagnostics is the development of simple and rapid tools to collect enough data for large clinical studies. Such data sets are necessary to identify novel biomarkers or correlations and to apply machine learning (ML) methods. It enables training of algorithms to recognize patterns in large datasets, which can be particularly advantageous for assays based on multiplexing. However, training requires extensive data sets from which the algorithms can learn. Optical biosensors based on nanomaterials fulfill the requirements for this development, providing the necessary sensitivity and specificity. A first key challenge is therefore to create variations of nanosensors in high throughput approaches.^[^ ^222^ ^]^ We believe that by combining different markers in a single test, patterns can be recognized and diagnosis simplified. This also includes the combination of specific and non‐specific sensors. The second challenge is to perform clinical studies that use such sensors and obtain the data required for ML.

Additionally, when developing a diagnostic test, it is essential to consider the target group. If the test is intended for everyday clinical practice, it must be as simple as possible and should not involve complex steps. This ensures its effective use in routine clinical settings. In summary, we believe that all these advances will lead to much more progress in the next years.

## Conflict of Interest

The authors declare no conflict of interest.
